# An Adjustable Magnetic Levator Prosthesis for Customizable Eyelid Reanimation in Severe Blepharoptosis II: Randomized Evaluation of Angular Translation

**DOI:** 10.1167/tvst.12.12.1

**Published:** 2023-12-01

**Authors:** Pooyan Tirandazi, Melanie Nadeau, Russell L. Woods, Eleftherios I. Paschalis, Kevin E. Houston

**Affiliations:** 1 Schepens Eye Research Institute, Massachusetts Eye and Ear, and Harvard Medical School, Boston, MA, USA; 2 University of Massachusetts Chan Medical School, Departments of Neurology and Ophthalmology, Worcester, MA, USA; 3 Central Western Massachusetts Veterans Affairs, Optometry Service, MA, USA; 4 Boston Childrens Hospital, Boston, MA, USA

**Keywords:** ptosis, prosthesis, magnetic

## Abstract

**Purpose:**

Examine the effect of force modulation via angular translation of a static magnetic field for customizable treatment of severe blepharoptosis.

**Methods:**

Prototype adjustable-force magnetic levator prostheses (aMLP) consisted of a spectacle-mounted magnet in rotatable housing and small eyelid-attached magnets embedded in a biocompatible polymer. Interpalpebral fissure (IPF) of 17 participants with severe blepharoptosis was continuously measured for one minute at five spectacle magnet angles, with order randomized and participant and data analyst masked. The hypothesis that angular position affected opening IPF (o-IPF), minimum blink IPF (m-IPF), and comfort ratings (1–10) was tested.

**Results:**

The aMLP improved o-IPF from 4.5 mm without the device to 6.2 mm on the lowest force setting (*P* < 0.001) and 7.1 mm on the highest setting (*P* < 0.001) and allowed for complete volitional blink regardless of setting (average m-IPF 0.4 mm and no change with aMLP; *P* = 0.76). Spontaneous blink without the device (2.0 mm) was affected on the highest force setting (m-IPF 3.9 mm; *P* < 0.001) but only marginally so on the lowest setting (3.0 mm; *P* = 0.06). Comfort (7.6/10) did not vary with the angle (*P* > 0.36). Profile analysis found substantial individual responses to angle (*P* < 0.001), confirming the value of customization.

**Conclusions:**

Angular translation provided adjustable force, which had a statistically and clinically meaningful impact on eye opening and the completeness of the spontaneous blink. This quantitative evidence supports continued use of the angular translation mechanism for force adjustment in the customizable magnetic correction of severe blepharoptosis.

**Translational Relevance:**

Evidence for the benefit of customizable magnetic force via angular translation in a larger sample of participants than reported previously.

## Introduction

Blepharoptosis, commonly abbreviated as ptosis, typically refers to abnormal eye opening with a low-lying upper eyelid margin. Ptosis may affect one or both eyes and be present at birth (congenital) or appear during later stages of life (acquired). Acquired ptosis occurs after damage to the third cranial nerve(s) or nuclei because of traumatic brain injury, stroke, viral illnesses, and diabetes. It can also occur due to pathology at the neuromuscular junction, as found in myasthenia gravis, or because of defects in the structure of the levator muscle or orbital contents as a result of injury, ocular surgery, or general aging mechanisms. When ptosis is severe, it obscures the visual axis and a large portion of the visual field, resulting in visual disability, similar in severity to that reported for patients with macular degeneration and retinitis pigmentosa (Sebastin, et al., Optom Vis Sci 2020;98: E-abstract 205203). Ptosis may cause functional issues because of a diminished superior visual field and interruption of primary gaze resulting in difficulty with performing daily tasks, such as reading, driving, and walking.^1^ In addition, it can cause indirect cosmetic-related deficits, such as looking tired or asymmetrical,^2^^,^^3^ which can decrease quality of life and increase anxiety.^4^ These issues are substantially worsened in cases of severe ptosis. Total bilateral ptosis causes profound yet potentially reversible visual disability.

Surgical intervention is the most common method to manage ptosis. Elevation of the upper eyelid is mainly facilitated by levator palpebrae superioris (levator) and superior tarsal (Muller's) muscles. In ptosis patients, when levator function is poor, elevation of the brow using the frontalis muscle can indirectly improve ptosis to some extent, making the frontalis sling the best option for severe cases.^5^ However, surgical correction of ptosis is not feasible for every patient and harbors risks, such as infection acutely or chronically from sling extrusion.^6^ In our experience nearly all sling patients experience some exposure keratitis, and in some conditions, such as chronic progressive external ophthalmoplegia, corneal erosions are common^7^^,^^8^ However, when overcorrection leads to corneal epithelial defects, there is a need for revision and, if not resolved, could lead to corneal scarring with permanent vision loss. In cases of variable ptosis, such as that in myasthenia gravis, surgery may be contraindicated. Also, the cost of surgery and need for highly trained surgeons is prohibitive in underdeveloped regions of the world.

Nonsurgical correction of ptosis has received less attention. These treatments usually involve a contact element by which the eyelid is pulled or pushed open. One of the first nonsurgical attempts to correct ptosis was described by Goldzieher in 1890,^9^ and later by others,^10^^–^^15^ is known as the “ptosis crutch.” This device uses a cushioned wire attached to the patient's spectacle to mechanically push the eyelid open. Another, more recent method is medical skin tape, used on the eyelid to pull the eyelid open.^16^^–^^19^ Although there is a paucity of data on safety and efficacy of the ptosis crutch or taping, from the limited reports and the authors’ experience, these methods appear to inhibit complete eye closure during blinks or require frequent adjustment, which often causes patient discomfort.^20^ Clinically, repetitive adjustment of the crutch may lead to eye injury. Similarly, recurrent application of the tape can cause lid skin and brow irritation. In extreme scenarios, the ptosis crutch can increase the risk of serious ocular damage in the event of impact, due to falling or being hit by an object. In 2020, oxymetazoline ophthalmic eye drops were approved by the United States Food and Drug Administration for mild age-related ptosis, but they only provide about 1 mm of additional opening^21^ and so would not provide enough improvement for moderate to severe cases. Use of an oversized scleral contact lens to prop the lid is another innovative approach previously described, but it is not easily adjusted.^22^^–^^26^

The use of static magnetic force is an attractive approach to treat ptosis. This method was first reported by Conway^27^ in the 1970s using ferrite magnets, which suffered from weak magnetic field and did not provide the necessary force to achieve full eye-lid opening, as needed in severe ptosis. More recently, Houston et al.^28^ developed a system using much stronger neodymium permanent magnets. This nonsurgical approach was named the magnetic levator prosthesis and was found to be effective and tolerable in participants with severe ptosis.^20^^,^^29^ Most importantly, the magnetic levator prosthesis not only restored eye opening but also allowed a volitional blink,^20^^,^^29^ and, unlike the ptosis crutch, there was no mechanical pressure on the eye or need for readjustment after each volitional blink.^20^ However, the prototype device used in these earlier studies interfered with the spontaneous blink.^20^ This limitation stemmed from the sensitive force distance relationship of static magnetic fields, which was titrated in those prior studies by adjusting the spectacle nose pads and by adding buffers (encasing the spectacle magnet to prevent strong adhesion). Buffers thickened the upper eye wire increasing the spectacle weight and, in some cases, obscuring the upper visual field, partially negating the benefit of ptosis treatment. Repeated adjustment of the fragile nose pads was not easily accomplished by the patient and could result in breakage, requiring frequent device repair. A better method for force titration may be clinically beneficial to account for differences in force needs to address factors between and within-patients, such as slight changes in lid magnet placement, frame movement, and the inherent variability of some types of ptosis, such as with myasthenia gravis.

Although force modulation could be achieved with electromagnets, they are heavier and more complicated, require a power source, and generate heat. A simpler approach would be to manually rotate the magnet on the spectacle frame, changing the polarity relationship between the lid and spectacle magnets. To achieve this, we developed a new magnetic levator prosthesis assembly frame that allowed angular orientation translation of the cylindrical spectacle magnet around its axis of polarization and thus manually delivered angular translation of the static magnetic field. Bench experiments during angular translation of a 12.7 × 12.7 mm cylindrical N-52 magnet produced approximately ± 3*g* force,^28^ which we hypothesized would be sufficient to provide useful effects on eye opening and completeness of eye closure during the spontaneous blink, in cases of severe ptosis. Because the volitional blink was intact even without adjustable force, the hypothesis was not relevant to volitional blinking. A recent study described bench force experiments and testing for design considerations in detail, and demonstrated proof of concept for this approach in two participants with severe blepharoptosis.^30^

In this study, we expand on that recent proof-of concept study by providing data for a larger sample of participants with severe blepharoptosis. This allowed for analysis of lid responses to and clinical utility of this novel adjustable force feature. We evaluated the hypothesis that different angular positions would be associated with significant changes in eye opening and closing as well as comfort level in ptosis patients, which varied (1) consistently across participants (group effect); or (2) differently within-participant (participant effect).

## Methods

### Fabrication Process and Materials

**Figure 1.**
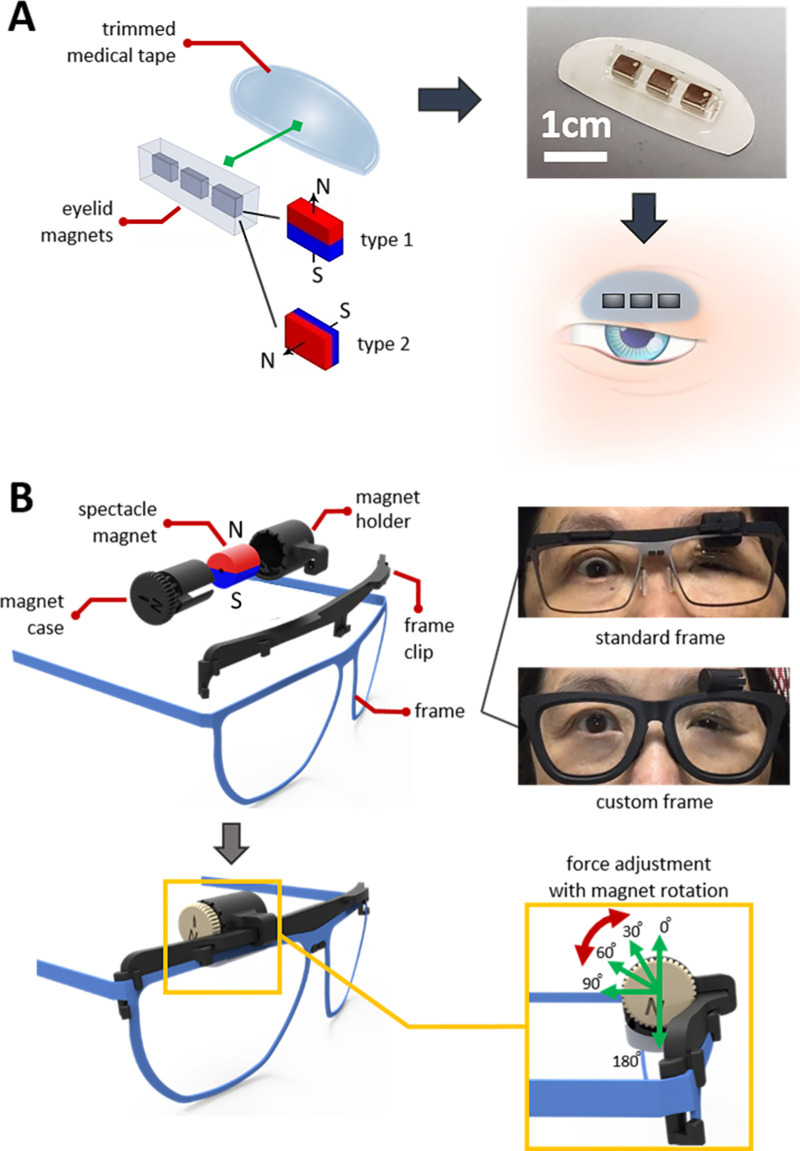
Schematic of the adjustable magnetic levator prosthesis parts. (**A**) Eyelid magnet layout and application. Two magnetizations were tested for the eyelid magnet; type 1 (N pole up) and type 2 (N pole out). (**B**) Spectacle magnet and frame with angular translation mechanism to change the magnetic field. Five angular positions were tested for this study. Two frame types were offered: a standard supplier frame with a mounting clip for the spectacle magnet and a three-dimensional–printed frame customized to each participant.

Figure 1 shows a detailed schematic of the components of the adjustable magnetic levator prosthesis. The adjustable magnetic levator prosthesis consisted of small magnets that were attached to the eyelid and a larger spectacle-mounted magnet that attracted the eyelid magnets which in turn lifted the eyelid to open the eye. We used permanent neodymium magnets (SM Magnetics, Pelham, AL, USA) 52 MGOe (1.44 T) for both the frame and eyelid magnets. The eyelid magnets were rectangular cubes (3 mm × 2 mm × 1 mm). Eyelid devices consisting of one, two, or three magnets were prepared by encapsulating the cubes in polydimethylsiloxane elastomer (Sylgard 184; Dow Corning, Midland, MI, USA)–fabricated with soft lithography and replica molding techniques. The polymer-magnet assembly was then bonded to transparent medical adhesive rolls (Opsite Flexifix; Smith & Nephew, London, UK) that were trimmed to the size and shape of the upper eyelid using a desktop cutting plotter machine (Cricut, South Jordan, UT, USA). The magnet-film assembly was externally applied to the eyelid skin (Fig. 1A). Two polarizations of eyelid magnets were tested, which we refer to as type 1 and type 2. When attached to the eyelid, the pole of the type 1 magnet was oriented vertically with north pointing up (relative to the gravitational plane), whereas the north pole of the type 2 magnet pointed out away from the eye, along the sagittal axis of the head.

The spectacle magnet was cylindrical (9.5 mm × 12.7 mm) and magnetized through its diameter (Fig. 1B). The magnet was inserted in a three-dimensional (3-D) printed enclosure on the spectacle frame that could be manually rotated with the fingers, allowing angular translation of the magnet around its axis to modulate the magnetic force (between the eyelid and spectacle magnets) (Fig. 1B). When wearing the adjustable magnetic levator prosthesis frame, the spectacle magnet sat near the eyebrow above the affected eyelid. Spectacle frames were produced as a 3-D printed clip-on magnet holder for a standard metal frame with adjustable nose pads (clinicians/participants chose from a few options) or as a 1-piece custom-sized 3-D printed frame (frame and magnet case all one piece; Skelmet, Boston, MA, USA).

### Study Design

The primary research question was whether adjustable magnetic force, via angular translation of the spectacle magnet through manual rotation (Fig. 1B), provided a measurable change in eyelid kinematics and may therefore be a valuable feature to incorporate. The study design was a prospective, double-blind, cross-over comparison of the effects of the spectacle-magnet angular positions on the interpalpebral fissure during eyelid open and volitional and spontaneous blink events. Our primary hypothesis was that angular position would be a significant factor that would affect the amount of eyelid opening and closing during the spontaneous blink, and participant-reported comfort level. Also, we hypothesized that the clinically selected best angular position would vary between participants, providing further support of the need for custom force adjustment as provided by manually adjustable angular translation.

### Study Procedures

Before the patient was fitted with the adjustable magnetic levator prosthesis, a visual acuity test, slit lamp examination with sodium fluorescein and lissamine green strips using the NEI-corneal staining scale, and one-minute baseline video were performed. Next, the eyelid was prepared with an eyelid scrub (Ocusoft lid scrub or similar) and dried. The eyelid magnet (polarization type 1 or 2, order balanced) was applied to the affected upper eyelid centered over the pupil as close to the lashes as possible. The adjustable magnetic levator prosthesis frame was placed on the participant with angular position of 0° (north-pole of the frame magnet facing up), 30°, 60°, 90°, or 180° (Fig. 1B). The order was approximately balanced, and participant and study staff, including clinicians, data processors, and statistician, were masked to the angular position. Comfort was surveyed with a Likert-type scale (most comfortable = 10) at each angular position. A one-minute video (iPhone 8; Apple, Cupertino, CA, USA) was recorded at each angular position, while the participant blinked naturally (spontaneous blinking). After one minute, the participant was asked to close and open their eyes three times to record volitional blinks.

### Clinical Judgment

The study clinicians, based on observations and feedback from the patient but masked to the angular position, selected the configuration of the adjustable magnetic levator prosthesis and angular setting that provided the best fit. Clinical study staff reported considering factors such as the amount of improved opening relative to the ability to blink, natural appearance of eyelid shape and the blink, eyelid apposition to the corneal surface, participant direct feedback, and participant nonverbal behaviors (e.g., increase blinking, squinting, grimacing, etc.). Then, based on the quality of eye opening and the blink, judged whether that fit met safety and efficacy standards, according to their clinical judgment, to continue with the device for an in-clinic trial of 20 minutes duration. The primary safety standard used by the clinical study staff was, at minimum, the ability of the participant to generate a complete volitional blink. Efficacy standard was related to the observation that the ptosis was improved and specifically that the visual axis was cleared. If favorable, an at-home wear trial was offered with approximately weekly follow-up visits. The results of extended use will be presented in a separate study. The present article will describe the results of eyelid response to angular translation during the short in-office sessions.

### Image Processing To Measure the Interpalpebral Fissure

**Figure 2.**
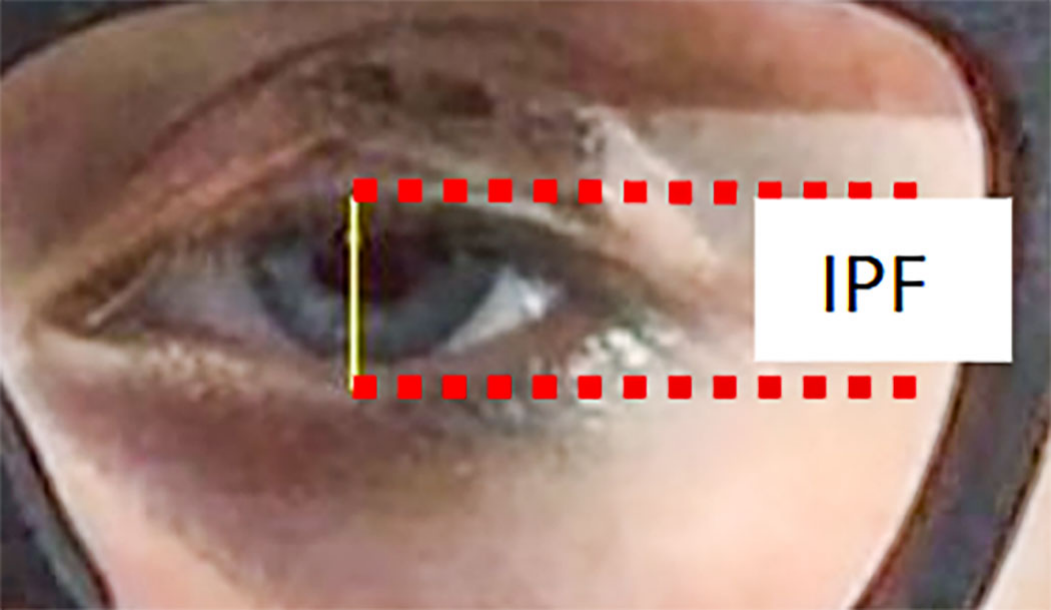
Frontal view of a participant with severe left eye ptosis corrected with the adjustable magnetic levator prosthesis. To quantify the amount of eye opening and closing, we measured the IPF, defined as the greatest distance between the upper and lower eyelid margins at the base of the eye lashes.

The 30 frames per second videos were converted to frames (stills) and the interpalpebral fissure was manually measured in every frame using ImageJ software (available at https://imagej.nih.gov/ij). Interpalpebral fissure, was defined as the greatest distance between the upper and lower eyelid margins at the base of the eye lashes as illustrated in Figure 2. The measurements were calibrated using the average horizontal visible iris diameter of 11.67 mm in the adult population, which is very consistent across gender and race with sample standard deviations of 0.32 mm^31^ to 0.42 mm.^32^ In a prior work, we found horizontal visible iris diameter to be at least as reliable as using a calibration marker on the forehead.^33^ Factors for analyses included spectacle-magnet angular position, eyelid-magnet type, frame type, and blink type (spontaneous or volitional).

**Figure 3.**
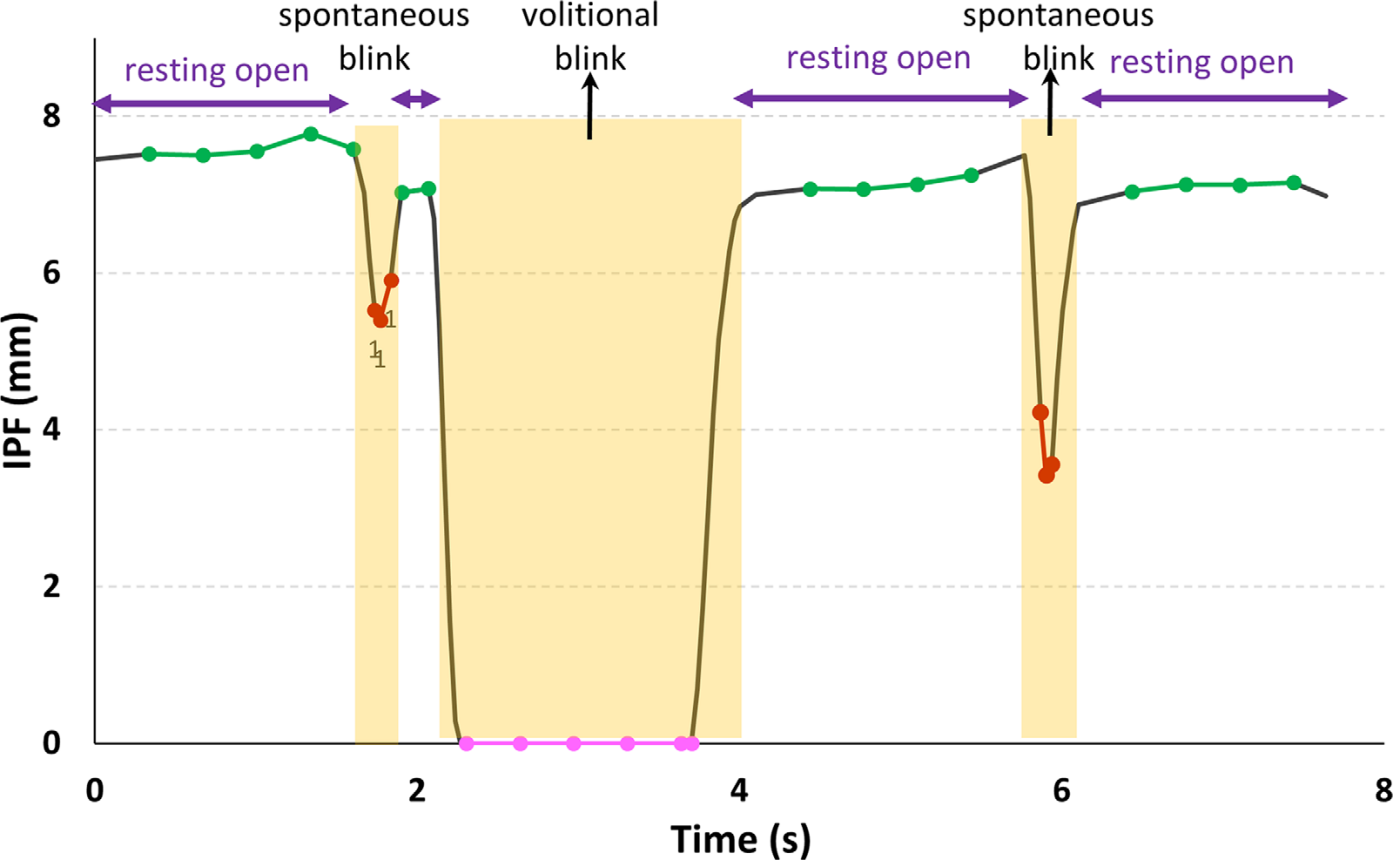
Example of an interpalpebral fissure (IPF) trace for a blink pattern that included two spontaneous blinks and one volitional blink. Highlighted regions represent the different blink periods; the horizontal arrows represent the span of the resting open periods also shown as the green segments of the trace; red circles are the three data points in the minimal IPF region of the spontaneous blinks; and the magenta diamonds represent that minimal IPF region of the volitional blink.

After frame-by-frame measurements, we plotted interpalpebral fissure against time to construct the blink pattern (trace) from each video recording, an example of which is shown in Figure 3. We separated the spontaneous blink and volitional blink periods, based on whether the participant was asked to perform a volitional blink or was allowed to blink naturally (spontaneous blink). Despite variation in the blink length between individuals and tasks, spontaneous blinks usually last a fraction of a second and may not normally be complete (full eye closure).^34^^,^^35^ Typically, volitional blinks lasted more than a second with a square-wave pattern (Fig. 3). To distinguish blink periods from eye-open periods, we employed an algorithm based on the first and second derivatives of the interpalpebral fissure trace to detect sharp falls (start of each blink cycle) and rises (end of each blink cycle) in interpalpebral fissure.^35^ For volitional blinks, we considered the interpalpebral fissure points between fortieth and sixtieth percentile of the data distribution within the blink period (magenta markers in Fig. 3). Limiting the included data to that within the middle of the within-blink period ensured that data points within the fast closing and opening phases of the blink (the sloped regions in Fig. 3) were not included. For spontaneous blinks, the three smallest interpalpebral fissure heights were used to represent the blinking interpalpebral fissure for that blink period (red circles in Fig. 3). This was selected over a single minimum point to reduce potential measurement biases and errors. Given the frame rate and blink duration, three points was the highest sample around the minimum that was consistently available for spontaneous blinks. Periods of resting open were defined using the distribution of all interpalpebral fissure points in the between-blink periods, with those that were within the interquartile region of 40th and 60th percentile being defined as being during resting opening (green markers in Fig. 3). This definition ensured that the eye-open points did not include the sloped regions (i.e., falls and rises) of blinks. A representative sample of interpalpebral fissure traces were manually reviewed and selected to have both typical blinks that were easily detected as well as those that were less obvious. Multiple criteria were tested and results were manually compared to the ground truth representative sample. Through this process an algorithm that produced full accuracy of blink detection in the representative sample was produced.

### Participants

The study followed the tenets of the Declaration of Helsinki and the protocol was approved by the institutional review board of Mass General Brigham (formerly Partners Healthcare). Informed consent was obtained from the participants after explanation of the nature and possible consequences of the study. Inclusion criteria were ptosis for one or both eye(s) which blocked the visual axis without action of the frontalis orbicularis muscle and with the head in neutral position; Mini-Mental State Examination^36^ score of 18 or better; and age ≥5 years.

Table 1 provides a summary of the 19 participants with severe unilateral or bilateral ptosis who enrolled in the study. Median age of the 19 participants was 51 years (range six to 85 years), and nine were female. Twelve participants had unilateral ptosis among which six had an affected right eye. One participant was screened and enrolled twice at different time points (S4 and S14, Table 1) but was ultimately removed from the study both times because of an impaired facial nerve causing an inability to overcome the force of the magnets to close the eye at even the lowest force setting. Another participant (S15, Table 1) was removed because of difficulties with communication and a self-inflicted corneal abrasion that occurred when not wearing the adjustable magnetic levator prosthesis. Therefore, 16 of 19 participants contributed data to the analysis. Across the many permutations of the study (spectacle-magnet angular position, eyelid-magnet type and frame type) there were missing data for particular conditions for multiple subjects due to fatigue from repeated testing and recovery between visits (data collection for the rotation experiment required two or three visits to complete), but this was handled adequately by the statistical models as shown by regression diagnostics reported in the results section. Incomplete data were tolerated by the statistical models without dropping any participants or conditions.

**Table 1.** Summary of the Participants

Participant ID	Age (Yr)	Gender	Ptosis Side	Severity (mm)	Cause
S01	28	M	Right	3.1 ± 0.05	CN III Palsy, TBI
S02	18	F	Left	4.8 ± 0.13	CN III Palsy, Brain Abscess
S03	78	F	Right	0.0	Stroke, Nuclear CN III
S04^*^	23	M	Right	N/A	Trauma
S05	41	F	Left	6.0 ± 0.14	CN III Palsy, Tumor
S06	60	M	Both	3.4 ± 0.19	OPMD
				3.8 ± 0.20	
S07	56	M	Both	5.3 ± 0.05	OPMD
				4.5 ± 0.05	
S08	28	F	Both	7.2 ± 0.04	CPEO
				8.5 ± 0.11	
S09	43	M	Left	4.6 ± 0.07	Congenital
S10	71	F	Left	0.5 ± 0.03	Sphenoid Wing Meningioma
S11	58	M	Both	6.2 ± 0.04	Top of Basilar Stroke, Nuclear CN III
				2.0 ± 0.04	
S12	55	M	Right	5.3 ± 0.06	Craniofacial Trauma
S13	85	F	Left	1.8 ± 0.04	CN III Palsy, Presumed CVA
S14^*^	23	M	Right	N/A	Trauma
S15^†^	6	F	Both	N/A	CN III Palsy, TBI
S16	47	F	Both	7.6 ± 0.17	CPEO
				6.8 ± 0.11	
S17	68	M	Right	0.0	CN III Palsy, Stroke
S18^‡^	55	F	Both	5.9 ± 0.04	CPEO
S19	17	M	Left	2.4 ± 0.05	CN III Palsy, Midbrain Pilocytic Astrocytoma

CN III, Cranial Nerve III; CPEO, chronic progressive external ophthalmoplegia; CVA, cerebrovascular accident; OPMD, oculopharyngeal muscular dystrophy; TBI, traumatic brain injury.

Severity: Defined as the mean baseline Inter-Palpebral Fissure during resting open. Severity of 0 mm is complete ptosis.

* S04 and S14 were the same participant enrolled twice but had to be removed due to persistent facial nerve weakness; not included in analyses.

† No useful data was available due to poor video quality.

‡ Participant had bilateral ptosis but sought treatment on one eye only.

### Statistical Methods

The effect of angular position was modeled using linear mixed-effects (multiple regression) models. The five angular (rotation) angles were randomly mapped over the arbitrary labels of 1 to 5 to mask the participant and study staff and were treated as categorical variables. The dependent variables (main outcomes) were the measured interpalpebral fissure or the reported comfort (separate models performed for each). The models for interpalpebral fissure included angular position fully crossed with eyelid blink state (blink event or resting open). Because there were repeated measurements on each eye and each participant might respond differently to each angular position, participant and angular position within participant-eye were included as random effects.

To model comfort level, we treated the reported Likert-type data as interval data and used a linear mixed-effect model with angular position number as the fixed effect and participant and angular position number within participant-eye as random effects. To statistically determine whether there were between-participant differences in response to angular position, we employed profile analysis which is a multivariate statistical technique that uses multiple analyses of variance for repeated measures to test piecewise parallelism. Demographic covariates, such as age and gender, did not have a meaningful effect on interpalpebral fissure or comfort, and thus, are not presented. Statistical analyses were performed using STATA 13 (StataCorp LLC., College Station, TX, USA) and R (R Core Team, 2017) with the lme4 library.^37^

## Results

### Adjustable Magnetic Levator Prosthesis Appearance and Fit

**Figure 4.**
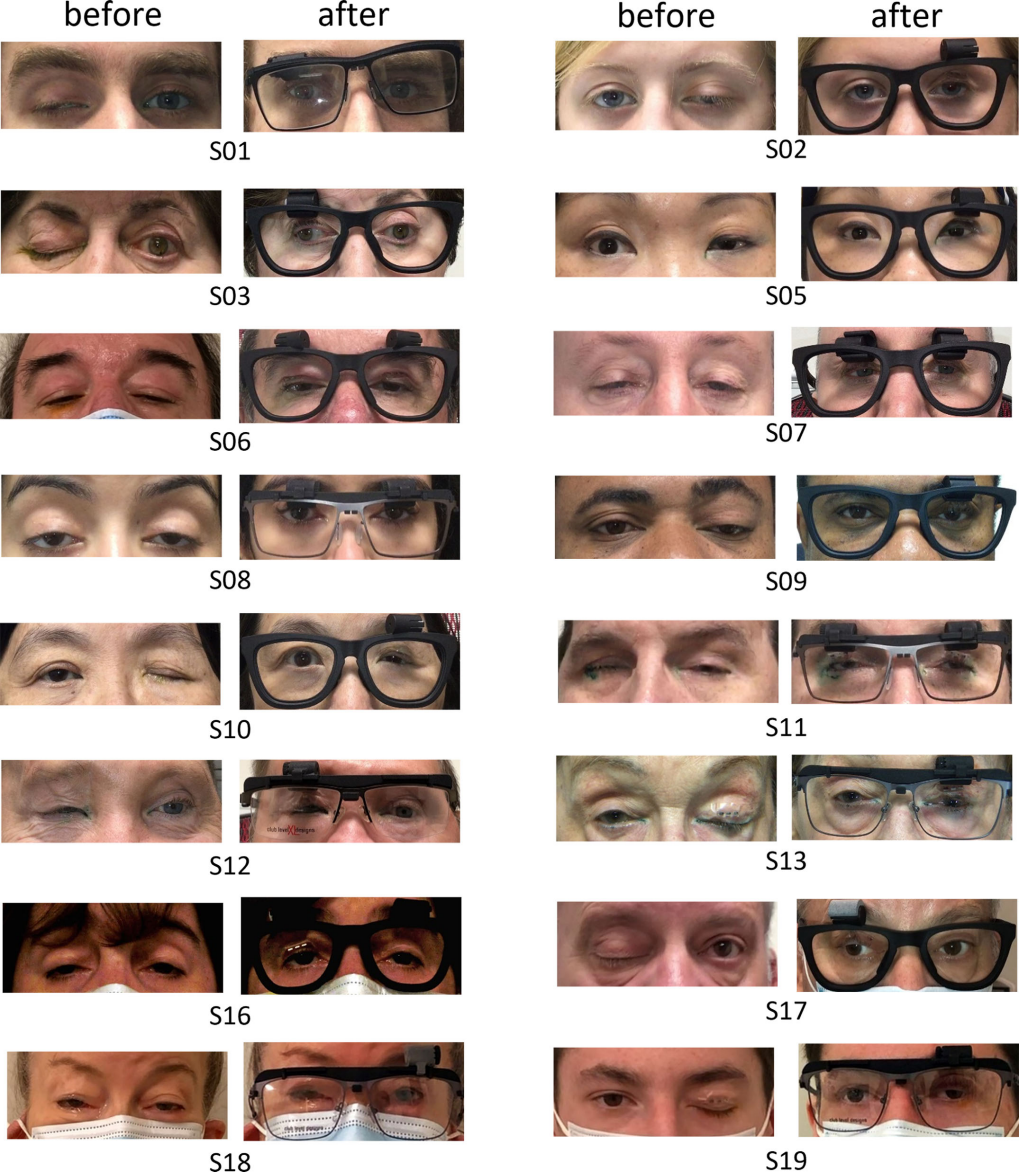
Frontal view snapshots of the participants at resting open without adjustable magnetic levator prosthesis (before) and when wearing adjustable magnetic levator prosthesis (after) at the position that was rated as best fit by the study clinician. An acceptable fit was not achieved for S08 because of poor fitting frames, and therefore the best possible opening condition is shown here. Images of S13 and S19 before the application of the eyelid magnet were not available.

Because this was a pilot study involving prototyping of the adjustable magnetic levator prosthesis, there were successive versions, described in detail in another manuscript.^30^ For each participant, the angular spectacle magnet position and eyelid magnet type that was rated as best-fit by the study clinician is shown in Figure 4. All participants, except S08, achieved a satisfactory fit. The best-fit angular setting was approximately evenly distributed across angles, as were the other fitting factors of eyelid magnet polarization type and frame type (Table 2). Every angular position tested was selected at least once as the best fit. The 180° angle was chosen more than any other angle followed by the 90° and 0°. Type 2 eyelid magnet (N pole out) was selected about 70% of the time (n = 12). The custom frame was selected over supplier frame 59% of the time (n = 10). The combination of type 2 magnet, custom frame, and 90° angular position was the most frequently selected configuration (about 30%). We note that, even with customized frames, the fit was still too large for S08 and S18. Having a loose frame sometimes caused the frame magnet and eyelid magnet to latch resulting in a wider opening than was desired and some resistance to closing.

**Table 2.** Counts of Clinician-Rated Best Fit Parameters

	Spectacle Magnet Angular Position					Lid Magnet Polarization		Frame Type	
	0°	30°	60°	90°	180°	Type 1	Type 2	Standard	Custom
Count of participant-eyes	4	3	1	4	5	5	12	7	10

**Table 3.** Counts of Participants by Rolling Direction Response Separated by Observed and Expected Responses

	0°	30°	60°	90°	180°	Total
Type 1						
Observed	9 min	4 min	3 out	5 out	6 out	27
Expected	10 min	7 min	8 out	8 out	8 out	41
Agreement	0.90	0.57	0.38	0.63	0.75	
Type 2						
Observed	11 out	7 out	3 min	7 in	11 in	39
Expected	12 out	12 out	11 min	11 in	12 in	58
Agreement	0.92	0.58	0.27	0.64	0.92	

Expected response was based on the predicted direction of rolling illustrated in Figure 9. Consistent with post-hoc predictions, torque behavior was quite consistent across the available sample for the 0° and 180° rotation angles; however, there was substantial variability at other rotation angles. “Min” in the table indicates that there was minimal rolling of the eyelid magnet.

### Spectacle Magnet Angular Position

**Figure 5.**
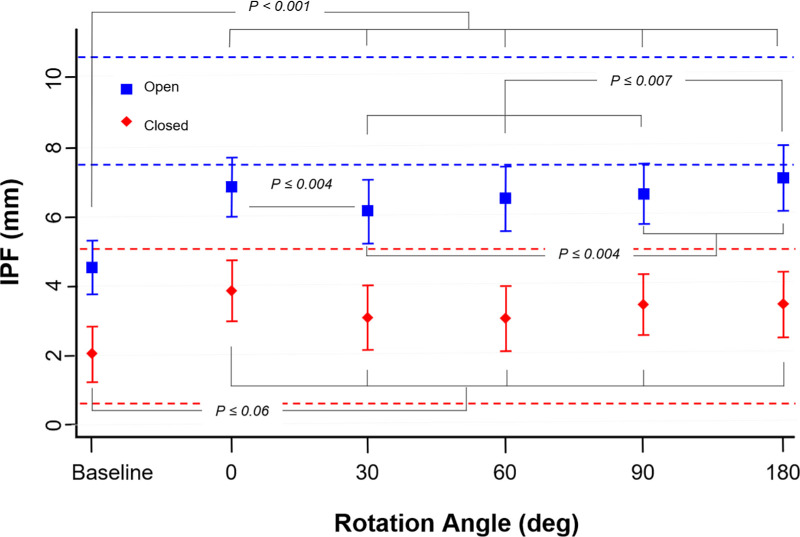
Regression model results (the means and 95% CI) for the effect of rotation angle. For eye-opening (blue squares), all rotation angles significantly improved ptosis from baseline. There was less opening for rotation angles 30° and 60° and more opening for 180°. For spontaneous blink (red diamonds), the amount of closing was marginally worse when wearing the adjustable magnetic levator prosthesis at any rotation angle. However, there was not a group effect of rotation angle (i.e., one rotation angle was not consistently [between subjects] better or worse than another). The horizontal dashed lines represent the 95% CI of the unaffected eyes for open (blue) and closed (red) states.

The five tested adjustable magnetic levator prosthesis angular positions were compared against not wearing a device (baseline), using a linear mixed-effects model. The fits to the model are illustrated in Figure 5. Compared to baseline without any device, the adjustable magnetic levator prosthesis significantly improved ptosis at any spectacle magnet angular position, from a baseline mean open interpalpebral fissure of 4.5 mm (95% confidence interval [CI], 3.8–5.3 mm) without device to 6.2 mm (95% CI, 5.2–7.1 mm; *P* < 0.001) on the angular setting with the lowest of amount of opening (30°) and 7.1 mm (95% CI, 6.2–8.1 mm; *P* < 0.001) on the angular setting with the highest amount of opening (180°). This put the open interpalpebral fissure with the adjustable magnetic levator prosthesis, on average, slightly below the 95% confidence interval from the open eye condition of the unaffected eyes (Fig. 5), mean 9.1 mm (95% CI, 7.6 to 10.6). In all angular positions, the adjustable magnetic levator prosthesis had some effect on the spontaneous blink minimum interpalpebral fissure (it decreased amount of eye closure) from a baseline without-device mean of 2.0 mm (95% CI, 1.2–2.9 mm) to 3.0 mm (95% CI, 2.1–4.0 mm; *P* = 0.06) on the lowest setting (60°) and 3.9 mm (95% CI, 3.0– 4.7 mm; *P* < 0.001) on the highest setting (0°). Note that the spontaneous blinks of the unaffected eyes also did not completely close (2.9 mm; 95% CI, 0.6–5.1; red dashed lines [Fig. 5]), as has been reported previously in healthy eyes.^38^

Volitional blink with the adjustable magnetic levator prosthesis was nearly always complete (mean 0.4 mm; 95% CI, −0.4 to 1.1 mm), meaning the participant could essentially always overcome the force to close the eye at will, and the eyelid closure on volitional blink was not different from without the device (*z* = 0.31, *P* = 0.76). When compared to the performance of the nonadjustable magnetic levator prosthesis prototype reported in a prior study (mean eye-open interpalpebral fissure 7.5 mm; 95% CI, 6.5 mm–8.5 mm),^20^ spontaneous blink was better (more complete) with the adjustable magnetic levator prosthesis.

Rotation angle of 180 degrees had larger interpalpebral fissure with resting open than the other rotation angles (z ≥ 2.68, *P* ≤ 0.007), except for rotation 0° (z = 1.08, *P* = 0.28), while rotation 30° had a smaller open-eye interpalpebral fissure than all other rotation angles (*z* ≥ 2.86, *P* ≤ 0.004), except for rotation 60° (*z* = 0.63, *P* = 0.53). Eye-opening and spontaneous-blink interpalpebral fissures were larger with adjustable magnetic levator prosthesis than without adjustable magnetic levator prosthesis (Fig. 5), there were no significant group differences between the angular positions (χ^2^(1) ≤ 0.54; *P* ≥ 0.46).

### Interparticipant Differences in the Intraparticipant Response to Rotation Angle

**Figure 6.**
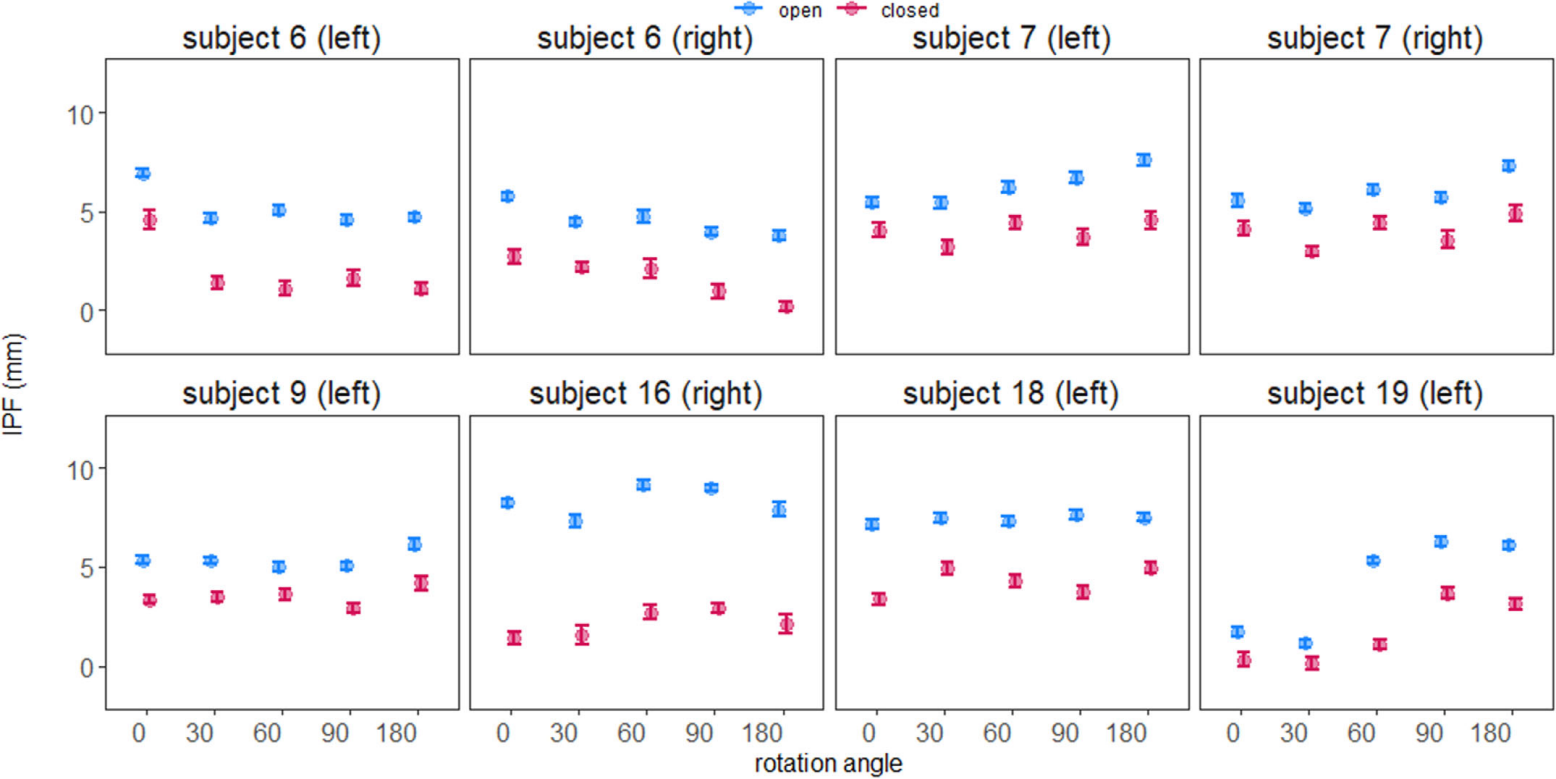
Participant-separated regression results of interpalpebral fissure (the means and 95% CI) at resting open and spontaneous blink for baseline and different adjustable magnetic levator prosthesis angular positions. For participants with bilateral ptosis the results are further separated by eye. We only show the participants for which a complete dataset of all five angular positions were available. All the participants were still able to perform complete volitional blinking regardless of the angular position despite incomplete spontaneous blink in some cases.

Based on observations during our review of the video blink recordings and individual participant data plots (Figs. 6 and 8), it seemed likely that rotation angle was affecting eye opening and blinking differently between participants. This meant that a rotation angle that resulted in better opening or spontaneous blinking in one participant was worse in another at a particular rotation angle. Figure 6 shows those different patterns of responses between participants, whereas Figure 8 shows a case example (S07) where there were clear differences in the blink kinematics in response to angular rotation. While some participants had little difference between the rotation angles (e.g., S06 left, S09, S16), others had rotation angles that were better than others (e.g., rotation angles 0° and 180° for participant S07, rotation angle 30° and 90° for participant S19).

This observation was in fact expected but the planned primary analysis could not address this type of response, because the participants were considered as random effects. To statistically evaluate this interparticipant variability, an alternative analysis was needed with participants as fixed effect in a profile analysis. Only the six participants (eight eyes) in Figure 6 for whom data was available at all five angular positions could be included. The profile analysis found that for both resting eye (*z* ≥ 7.83; *P* < 0.001) and spontaneous blink (*z* ≥ 6.87; *P* < 0.001) indeed the between-subject differences were statistically significant. These individual differences in the effect of the rotation angle indicate that there was no single best rotation angle when considered at the patient or eye level, confirming the benefit of customization via the adjustable force feature.

### Comfort Rating

**Figure 7.**
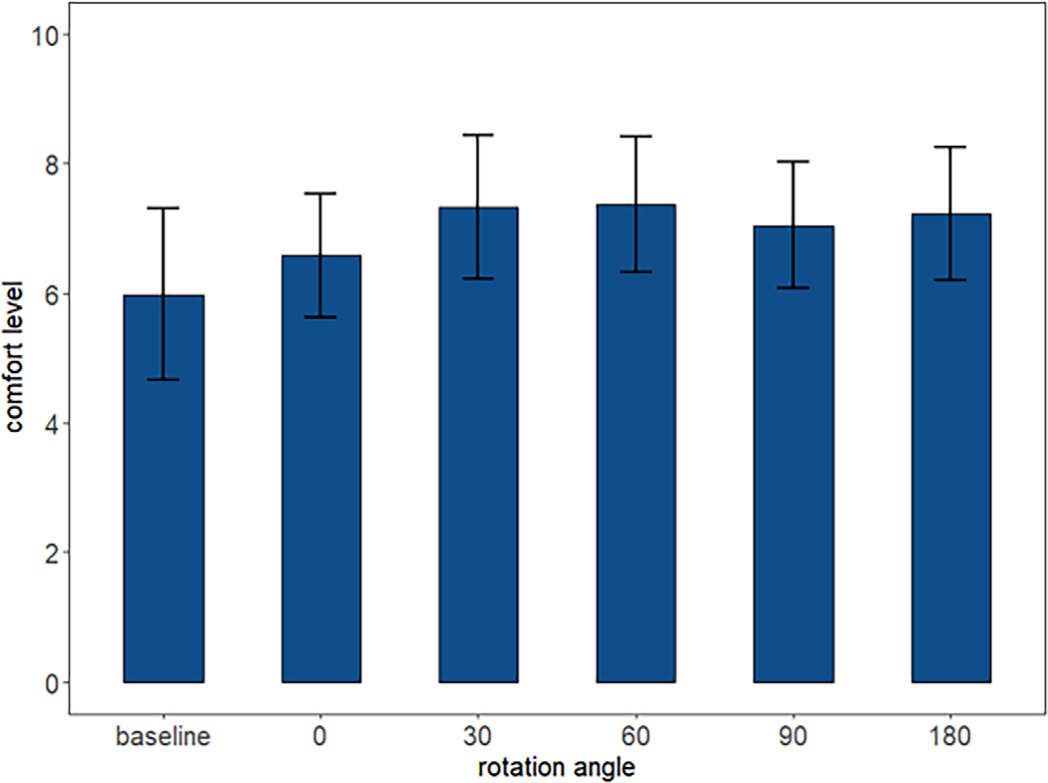
Regression results of reported comfort level (scale of 1 to 10 from worst to best, the means and 95% CI). Except for 0°, reported comfort was higher with adjustable magnetic levator prosthesis angles than the baseline. However, the group differences in comfort by rotation angles were not significant.

The mean baseline comfort rating without any device was 6.0 out of 10 (95% CI, 4.7–7.3). There were trends for reported comfort to improve with the adjustable magnetic levator prosthesis for all rotations (*z* ≥ 1.65, *P* < 0.10) except 0° (*z* = 0.94, *P* = 0.35) (Fig. 7). The differences in comfort between rotations were not significant in our sample (*z* ≤ 1.63, *P* > 0.10).

### Post Hoc Evaluation 1: Eyelid Magnet Torque Behavior When Varying the Spectacle Magnetic Field

**Figure 8.**
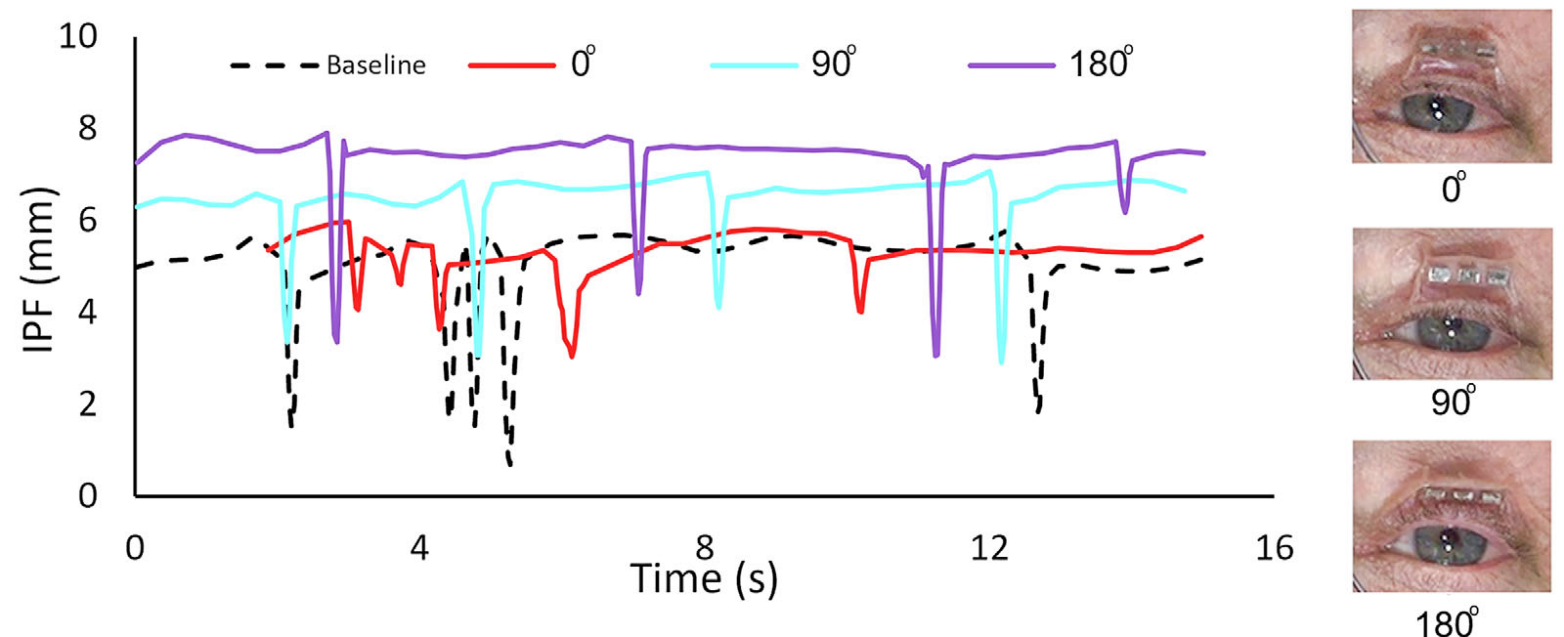
Example interpalpebral traces and video frame captures for the left eye of participant 7, with type 2 eyelid magnet (north pole out), showing the representative beneficial effect of varying orientation of the magnetic field by 0° to 180°. Note that the spectacle magnet, which is not visible in the cropped video frame but sits directly above the eyelid magnet, was oriented with north pole being up at 0° and down at 180°. For this participant, 180° was the best with the most opening yet similar closing on spontaneous blink (spikes in trace data). Volitional blinks are not show in this trace segment, but were complete for all rotation angles.

**Figure 9.**
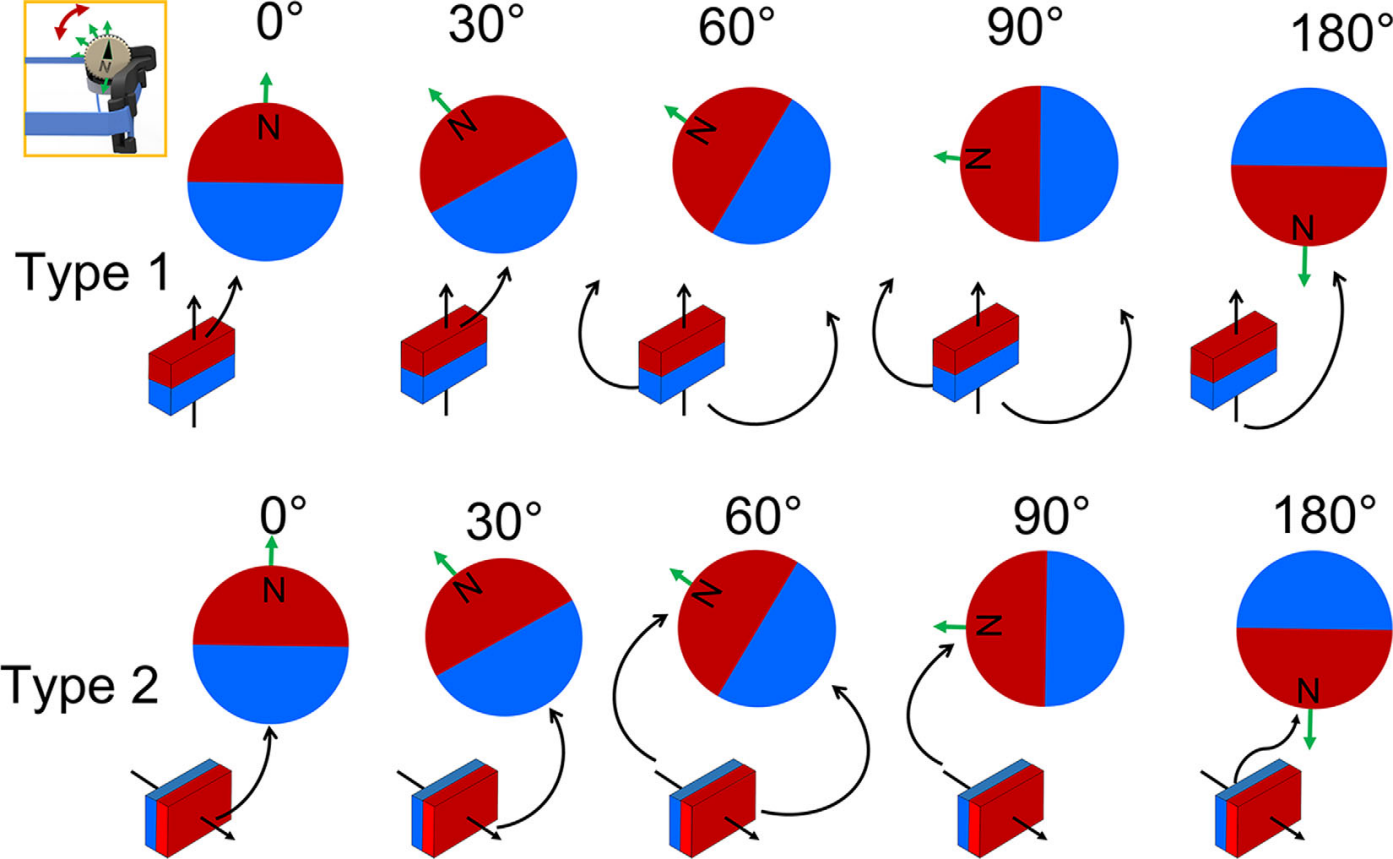
Predicted array torque and translational vectors for the tested spectacle magnet orientations, when assuming a posterior displacement of the lid magnet relative to the spectacle magnet (positive back vertex distance). Only one of the three eyelid magnet cubes was drawn for ease of visualization and was not drawn to scale. At 60° and 90° rotations, direction of eyelid magnet rolling would have been transitioning from rolling in or out and so was less predictable.

**Figure 10.**
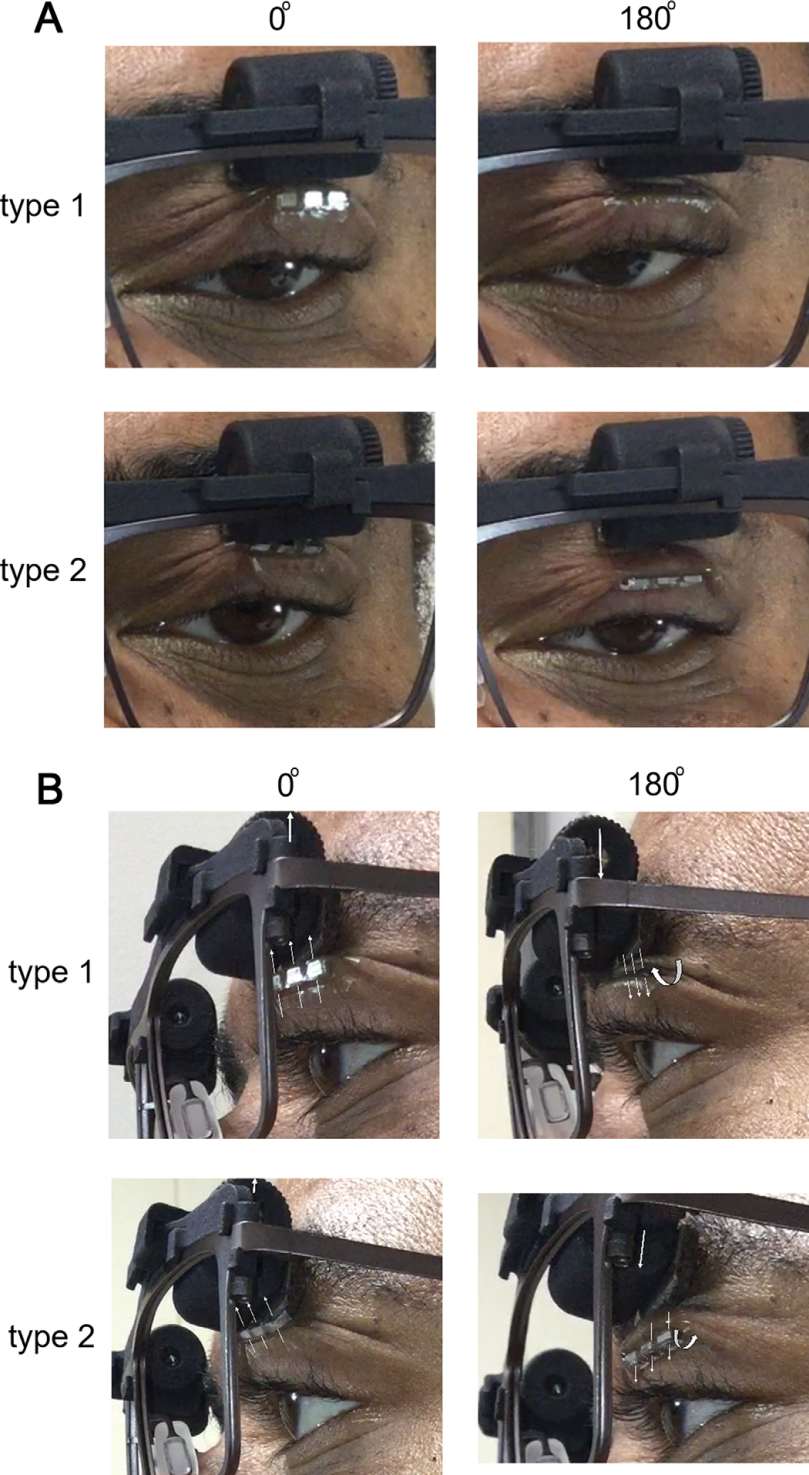
(**A**) Frontal and (**B**) sagittal view of participant S09 providing examples of eyelid magnet rolling based on spectacle magnet angular rotation and eyelid magnet polarization type. White arrows denote the direction of the north poles. When the spectacle magnet was set to 0°, type 1 eyelid magnet did not roll, whereas type 2 rolled outward (lower edge away from the eye). In such case the eyelid magnet directly contacted the spectacle magnet casing (the buffer), where forces were relatively high. When the angle was set to 180°, eyelid magnet rolled inward for type 2, and outward for type 1. Notice that the rolling helped to conceal the eyelid magnet and acted as an additional buffer in both configurations but the contact between the eyelid and the ocular surface was best in the type 2–180° configuration for this participant.

In the fitting process, the clinician study staff observed torque behavior of the eyelid magnets for certain orientation positions. This is conceivable since the magnetic poles were not always aligned, creating magnetic torque flux, which could result in the eyelid magnets rolling to align the poles as schematically illustrated in Figure 9 and photo documented in Figures 8 (lower panel) and 10. Factors influencing the amount of rolling were clinically interpreted as being related to skin laxity, placement and other mechanical factors. To explore the observations and predictability of rolling behavior, we performed a post hoc comparison of the direction of rolling expected based on the schematics in Figure 9 to that observed in the video recordings, per example in Figure 10 observed and expected with levels of agreement are shown in Table 3. For type 1 (North pole up), expected torque behavior was for the bottom of the magnet to show minimal rolling in the 0° or 30° positions and begin to show outward rolling at 60° and 90° with complete inversion via outward rolling at 180°. For type 2 eyelid magnet (N pole out), the expected torque behavior was for the bottom of the eyelid magnet to roll out away from the eye in the 0° and 30° setting, minimal to no rolling in the 60° setting, and inward in the 90 and 180° settings. Note that expected rolling direction assumed a positive back vertex, i.e. the eyelid magnet having linear displacement posteriorly (closer to the eye) than the spectacle magnet, as is evident in the sagittal camera angle in Figure 10B. For both eyelid magnet types, the torque behavior was quite consistent across participants for the 0° and 180° but not the other rotation angles. For type 1, 90% of participants exhibited the predicted no rolling behavior at 0° and 75% showed the predicted outward rolling at 180°. For type 2, 92% of participants showed the predicted outward rolling at 0° and inward rolling at 180°. The lowest levels of agreement between expected and observed were for the 30° and 60° orientation (57% and 38% type 1 and 58% and 27% type 2), which is when the direction of rolling would have been transitioning. There was substantial variability in the response to these 30° and 60° rotation angles and therefore the ability to customize the force for each participant is preferable. Possible reasons for the variability may be related to between-participant variance in skin laxity, orbital structure, or back vertex distance.

### Post Hoc Evaluation 2: Eyelid Apposition

**Figure A1.**
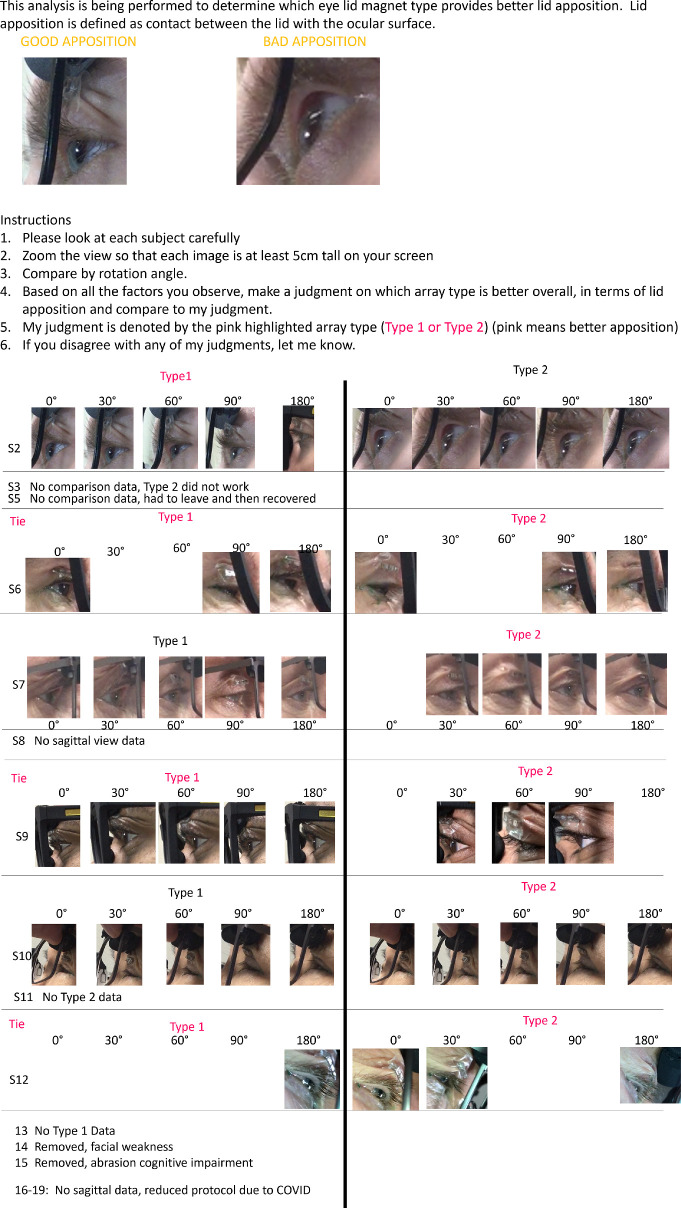
Frames of sagittal videos reviewed to evaluate for poor lid apposition and possible relation to spectacle x lid magnet polarization orientation.

A likely important observation by the clinical study staff was poor apposition of the eyelid to the globe in some participants which was modifiable by changing spectacle magnet orientation or eyelid magnet type. To explore this observation, sagittal videos were reviewed by two of the authors (K.H. and M.N.) across the rotation angles and judgments were made as to whether there was poor apposition, and for which array type there was better apposition. This video was difficult to acquire as the view could be obscured by the frame temple or lost with movement of the participant, and so data was not available for many participants. During data collection during and in the year after the COVID-19 pandemic in 2020, study visits were shortened to include only critical elements, and so sagittal videos were not taken for S16 through S19. In total there were at least partial data for six participants (enough to judge apposition and response to force adjustment), and complete data for 3 participants (see Appendix Fig. A1 for frame captures of the videos reviewed). Poor eyelid apposition was present for at least one condition in four of six participants. For all four the issue was resolved by adjusting to a different spectacle magnet orientation or eyelid magnet type. One participant had better apposition with type 1, two were better with type 2, and 3 had equal response. We concluded that the study clinicians’ observations were correct that poor lid apposition occurred and could be successfully modified by force adjustment; however, there was not a clear difference between array types in this sample and eyelid apposition may be more related to contact between the eyelid and spectacle magnets.

## Discussion

Our findings support the primary hypothesis that angular translation of the static magnetic field via a manual dial on the adjustable magnetic levator prosthesis frame was a simple yet effective method to provide adjustable force. Significant changes in eyelid behavior in response to the angular position of the spectacle magnet were measured both for the amount of eyelid opening and the completeness of the spontaneous blink.

Objective data was supported by clinical study staff reports concerning the value of the angular translation feature. The adjustable magnetic levator prosthesis provided superior performance for spontaneous blink compared to the non-adjustable Magnetic Levator Prosthesis prototype reported previously.^20^ However, the completeness of spontaneous blink was still significantly worse than in the unaffected eye of unilateral cases of this present study, leaving room for further improvement.

Both the clinically selected best angular position and objective lid kinematic data varied between participants, providing strong evidence for the importance and value of customizing the configuration. Even with individual differences (Fig. 6), there were some significant group trends. An increase (improvement) in opening was usually accompanied by increase (worsening) in the completeness of the spontaneous blink (Fig. 6). However, some settings did expand the interpalpebral fissure range between opening and spontaneous blink events, providing better opening and better spontaneous blink, or better opening without worsening of spontaneous blink. Overall, the group data suggested that 0° performed well for opening but compromised the performance of spontaneous blink. Conversely, 90° provided better blinking compared to 0° and 180°, but was not the best option for maximum opening. Perhaps the most unexpected and interesting finding was the behavior of the lid magnet in the translated field, which counter-rolled via magnetic torque flux to realign the poles rather than producing repulsion. We think this occurred because the north and south poles are relatively close together in the eyelid magnets; therefore, the propensity to realign was high. Our post-hoc analyses found the direction of rolling was highly predictable for the 0° and 180° spectacle magnet orientations, but substantial individual variability occurred at other rotation angles. The amount of rolling seemed to be influenced by the eyelid skin laxity–an as yet non-quantifiable factor influencing performance. As such, the configuration most often selected as “best” (by clinician/participant) was with the spectacle magnet set to reversal (180°) when in the type 2 eyelid magnet polarization type (polarization in the sagittal plane, as worn by participant S07 in Fig. 10), which rolled the lower edge inward. This folded the skin between the eyelid magnet and spectacle magnet, effectively opening the eye but keeping a larger separation at the peak of opening, with less force to overcome for spontaneous blink. The direction of the forces appeared different in this configuration as well, perhaps moving more inward and back toward the eye to more closely simulate the action of the defective levator neuro-muscular complex. Unfortunately, the available video data was not sufficient to objectively measure and confirm this hypothesis. It is worth noting that depending on the eyelid skin thickness and laxity, the rolling behavior could increase eye opening by different amounts. In participants with less laxity from thicker or more taut skin, the eyelid magnet did not seem to roll as much, presumably due to mechanical restriction, with less eye opening.

The analysis of eyelid magnet polarization type did not provide a clear trend for which was better overall and was not included in the main manuscript due to poor regression diagnostics. This analysis is provided in a supplement. In that data, type 1 eyelid magnet polarization (north pole up) provided better opening at most angles; however, it was preferred less by clinical judgment (Table 2), perhaps due to appearance or quality of the blink.

### Limitations

There were limitations of the current study. While there was a large volume of quantitative data and our primary outcomes were robust and thus unlikely to be affected substantially by additional enrollment, the sample size was fairly small which limits our ability to confidently rule out significant secondary factors such as age and gender. There is also the possibility that our sample was not representative of the larger U.S. severe ptosis population, limiting generalizability of results. The full five angular positions were only tested at the immediate time point; therefore, relative performance over longer durations was not analyzed. Participants in this study were offered participation in a study extension wherein they did an at-home use trial for a median of eight weeks, which will be reported in a separate study. However, this at-home use arm did not include repeat evaluation of the spectacle magnet orientations (they were only recorded with the setting they found to be best for their needs).

The adjustable magnetic levator prosthesis is not a commercial product and is not available for clinical use at this time, and so clinical practice will not be immediately changed by the results presented here. A report on an ongoing double-blind randomized clinical crossover trial will follow the study reported here. That study is using the adjustable force feature in the study device and comparing that adjustable magnetic levator prosthesis device to kinesiotaping of the ptotic eyelid(s). That study will answer additional questions concerning longer term safety, generalizability, and noninferiority to the main existing non-surgical option (taping of the eyelid). Safety concerns that could result from at-home use of the device, such as lid skin irritation or interaction of the magnetic glasses with electronics, magnetic resonance imaging, orbital and facial reconstruction hardware, or implantable cardioverter-defibrillators (ICDs) have been considered and are being monitored for issues in prior and ongoing chronic use studies. For orbito-facial reconstruction, mostly titanium is used which is paramagnetic. There is one in vitro report that common neodymium magnets, such as those off a fridge or in electronic devices, could theoretically affect older ICDs, but there are no cases in the clinical literature reporting a safety issue.^39^^,^^40^ Participants with ICDs are advised not to allow the MLP to contact their chest near the pacemaker site as a precaution. Newer ICDs are even less impacted or not at all, and some are rated as magnetic resonance imaging safe. If results with the adjustable magnetic levator prosthesis continue to be positive, it may be commercialized by a medical device company, but at the time of writing this there were no plans to do this.

For future studies we recommend higher spatial resolution video recording, which is expected to allow better automation of interpalpebral fissure measurements, thereby reducing the burden of manual measurement used in this and prior studies of the magnetic levator prosthesis. A side sagittal-view iPhone camera was used in this study, but resolution was not ideal. Future studies may simply have the patient turn their head left, right, up, and down at the end of each frontal view recording. An integrated multi-camera system could also be useful to produce a 3-D model of the blink kinematics relative to the 3-D positioning of the magnetic elements. Such techniques may reveal additional factors that influence eyelid behavior, potentially leading to further improvements in design and the completeness of spontaneous blink.

## Conclusions

There were significant effects of angular static magnetic field manipulation at both the group and individual levels, supporting a clinically meaningful effect using this method of force adjustment and direct evidence for the value of customization. The adjustable magnetic levator prototype in this study showed similar efficacy for eye-opening compared to the prior study with a nonadjustable version; however, the adjustable version here had improved spontaneous blink when individually adjusted by the study clinicians. Because there were clear differences between subjects in their response to the angular positions, the angular position mechanism provided the ability to titrate the force and thereby provide a customized fitting, which is likely to enhance usability in the clinical setting.

## Supplementary Material

Supplement 1

## Appendix

Two authors (K.H. and M.N.) reviewed high magnification sagittal video frames and made consensus judgments on lid apposition by lid magnet type.
