# Supplementary material for: An Adjustable Magnetic Levator Prosthesis for Customizable Eyelid Reanimation in Severe Blepharoptosis II: Randomized Evaluation of Angular Translation
Source: Transl Vis Sci Technol. 2023 Dec 1;12(12):1. doi: 10.1167/tvst.12.12.1 (PMC10697171; doi:10.1167/tvst.12.12.1)
Supplement: Supplement 1 [file tvst-12-12-1_s001.docx]

## **Supplement 1 (S1):** Effect of Eyelid Magnet Polarization Type

S1 Methods

To model the effect of eyelid magnet type, we added the magnet type variable and its interaction with angular position as fixed effects. To examine whether there were individual differences in responses to angular position, we used the data for the eight eyes with data for all five angular positions to conduct a profile analysis. This involved a repeated-measures ANOVA and a post-hoc test of parallelism.

S1 Results

Averaged across all rotation angles, both open interpalpebral fissure (z=9.58, p<0.001) and spontaneous blink interpalpebral fissure (z=14.7, p<0.001) were greater with type 1 (north pole up) than type 2 (north pole out). Further, there were interactions between polarization type and angular position (Figure ). In particular, the combination of type 1 and 60° had a large interpalpebral fissure on spontaneous blink (5.7mm), there was no difference between polarization types for 90° (z≤1.22, p≥0.22), and polarization type 2 had a larger interpalpebral fissure on spontaneous blink than type 1 for 30° (z≥6.22, p<0.001).

| 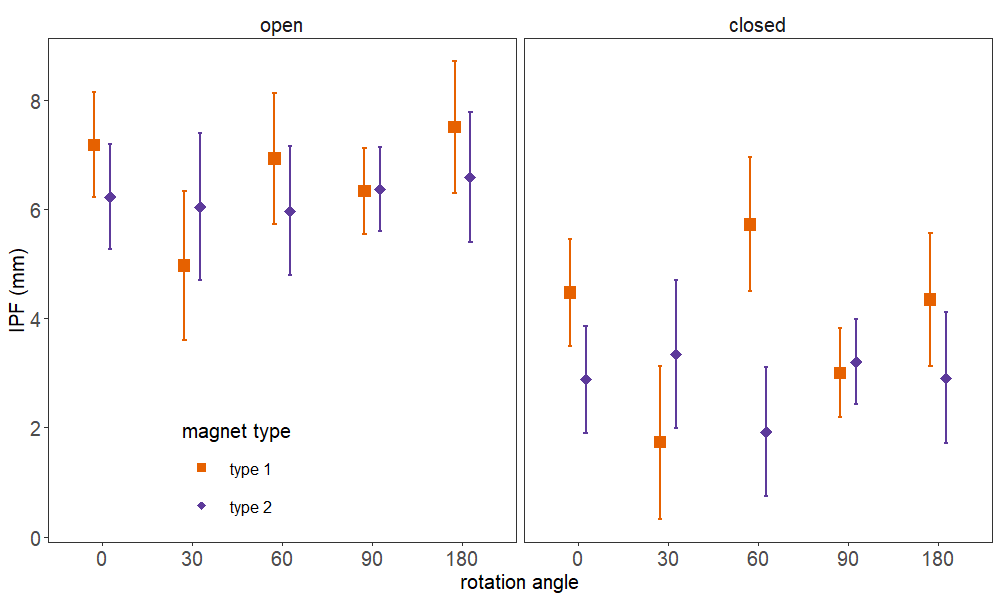 |
| --- |
| Figure S1. Comparison between the regression results (the means and 95% CI) of eyelid magnet polarization type at different angular positions on INTERPALPEBRAL FISSURE during resting open and spontaneous blink (closed). In general, both types provided significant opening but type 1 magnets had better opening performance (larger INTERPALPEBRAL FISSURE), while type 2 had better spontaneous blink performance (smaller INTERPALPEBRAL FISSURE. Note the large difference between type 1 and 2 for the 30 and 60 degree spectacle magnet orientations. At 60 degrees type 1 had poor spontaneous blink closing and at 30 degrees type 2 had much poorer closing. |

S1 Discussion

The analysis suggests, that Type 1 provides greater opening but also worse s-blink. It also suggests that for s-blink, at 60 degrees, there was a huge difference between type 1 and 2, and it changes with the next setting of 30 degrees. At 60 degrees Type 1 had poor spontaneous blink closing and at 30 degrees type 2 had much poorer closing. In order to try to understand this finding we conducted a detailed review of the data and found that this secondary analysis was based on only 2 complete data sets. The rest of the data was for subjects who did not have all the rotation angles or were missing one condition entirely. We also reviewed all the videos of blinking to try to understand if the finding was interpretable or if a reasonable hypothesis could be formed (See Figure S1). After many hours of inspection, we could not be confident that the finding represented a real effect and so removed it from the main body of the manuscript. It is being provided as a supplement so that the data can be compared with any future studies that may examine these eyelid magnet polarization orientations.
